# Implications for applicability of the photodegradation and self-recovery of green-emitting CsPbBr_3_ perovskite nanocrystals †

**DOI:** 10.1039/d4ra04567j

**Published:** 2024-08-19

**Authors:** Yoshiki Iso, Shunsuke Saito, Hikari Toyoda, Tetsuhiko Isobe

**Affiliations:** a Department of Applied Chemistry, Faculty of Science and Technology, Keio University 3-14-1 Hiyoshi, Kohoku-ku Yokohama 223-8522 Japan iso@applc.keio.ac.jp isobe@applc.keio.ac.jp +81 45 566 1551 +81 45 566 1558 +81 45 566 1554

## Abstract

CsPbBr_3_ nanocrystals (NCs) synthesized by the conventional hot-injection method are photochromatic luminescent nanomaterials due to the photoinduced desorption and re-adsorption of the surface ligands. The apparent color and the luminescence intensity were changed significantly during excitation light irradiation and following dark storage; however, the emission wavelength was almost retained. This work investigates the change in emission color of light-emitting diode lighting using the CsPbBr_3_ NCs to realize photochromatic luminescence. The results showed definite shifts in emission color caused by changes in optical absorption and green luminescence intensity of the NCs, potentially broadening the application feasibility of CsPbBr_3_ NCs as photochromatic luminescent nanomaterials.

## Abstract 2

The photodegradation and self-recovery of CsPbBr_3_ nanocrystals changed body color, luminescence intensity, and illumination color of devices, potentially broadening applicability.

## Graphical abstract

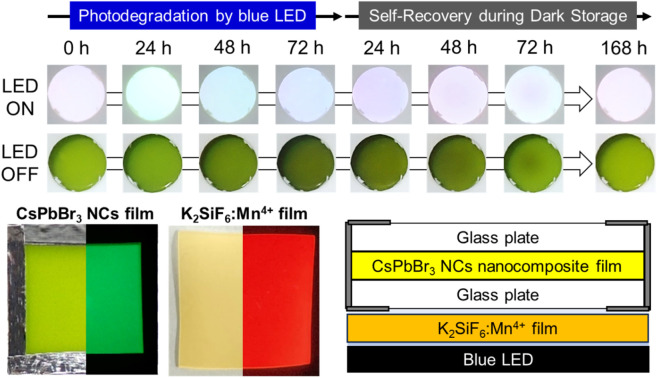

## 1 Introduction

Colloidal CsPbX_3_ (X = Cl, Br, I) perovskite nanocrystals (NCs) have attracted great interest in various fields due to the fascinating luminescent properties.^1^ There are many reports on their synthesis, hybridization, stability, and applications including sensors, biological imaging and optoelectronic devices such as light-emitting diode (LED) lighting and wide color-gamut displays.^2–6^ Surface ligands adsorbed on CsPbX_3_ NCs have a significant impact on colloidal stability, structural stability, and optical properties.^7,8^ We previously found and reported photodegradation and self-recovery of CsPbBr_3_ NCs.^9–11^ When the dried solid sample was irradiated with excitation light, the color changed from yellow to black and the photoluminescence (PL) intensity decreased at the same time. The color and PL intensity of the degraded sample naturally returned to the initial state during the dark storage after the excitation light irradiation was stopped. Based on the Fourier-transformed infrared (FT-IR) absorption measurement, the photodegradation is attributed to the generation of surface defects due to photo-induced desorption of the surface ligands, while the self-recovery in the dark is attributed to surface passivation due to their re-adsorption. The change in PL lifetime also occurred parallel to the change in the FT-IR absorption. The PL lifetime was shortened by photo-excitation, which is attributed to the non-radiative relaxation through surface defects generated by photoexcitation. In contrast, the PL lifetime was completely recovered during the dark storage, which is attributed to the elimination of surface defects by surface passivation.^9^

In the field of perovskite-based photovoltaics, rapid self-recovery of photodegraded polycrystalline perovskite layers within 1 min has been reported. Its mechanism could be explained by ion migration.^12,13^ This phenomenon is too fast and would make its own application difficult. In contrast, the self-recovery of photodegraded CsPbBr_3_ NCs occurred slowly due to re-adsorption of surface ligands. This is a distinguished phenomenon for the NCs adsorbed with surface ligands; it would possibly realize new applications due to the easier recognizability by humans.

One of the main features of CsPbX_3_ NCs is that the halogen composition controls the bandgap and the emission color. The self-recovery after photodegradation was observed even when Br was partially replaced by Cl or I.^11^ The change in sample color under excitation light is photochromism. Photochromic materials have a wide range of potential applications in molecular switching, detection, biology, and molecular mechanics.^14^ Photochromism is generally caused by structural changes in organic dyes. Many hybrid materials with inorganic crystals have been reported;^14^ however, there are only few reports on inorganic nanomaterials such as Ag nanoparticles and Cu-doped ZnS.^15,16^ Hybrid photochromic materials based on CsPbX_3_ perovskite have also been investigated; photochromism was intrinsically realized by structural changes of organic molecules.^17,18^ The photochromism of CsPbX_3_ NCs that we have been investigated on is due to the formation and decrease of surface defects, which is an unusual phenomenon with a very different mechanism. Our photodegradation and self-recovery cause a large decrease and recovery of PL intensity, while the emission wavelength does not change much. Photochromic PL switching is a technology that has attracted much attention in recent years.^19^ If not only the sample color but also the emission color can be changed simultaneously, it is expected to be used for a wider variety of applications. We therefore focused on mixed light color of the green PL of the NCs and blue excitation light.

In this work, a nanocomposite film of CsPbBr_3_ NCs was placed on a blue LED to fabricate a lighting device. The light observed from this device is a mixture of green PL and transmitted blue from the LED. The apparent emission color is thought to change with photodegradation and self-recovery of the CsPbBr_3_ NCs in the green PL layer. Furthermore, a white lighting device was fabricated using a red phosphor, K_2_SiF_6_:Mn^4+^ (KSF), which is a practical material used in wide color gamut displays.^20,21^ Changes in the luminescent color of the lighting devices were evaluated during continuous exposure to blue light and during the subsequent dark storage without LED illumination.

## 2 Experimental

### 2.1 Materials

Cs_2_CO_3_ (99.99%, Mitsuwa Pure Chemical), PbBr_2_ (99%, Mitsuwa Pure Chemical), and granular ethylene-vinyl acetate (EVA) resin (Evaflex EV150, Du Pont-Mitsui Polychemicals) were used as received without further purification. 1-Octadecene (>90.0%, Tokyo Chemical Industry), oleic acid (OA; >85.0%, Tokyo Chemical Industry), oleylamine (OAm; 80–90%, Acros), *tert*-butanol (>99.0%, Tokyo Chemical Industry), and toluene (>99.5%, Kanto Chemical) were dehydrated over molecular sieves (3A1/8, FUJIFILM Wako Pure Chemical Industries) prior to use.

### 2.2 Preparation of Cs-oleate precursor

CsPbBr_3_ perovskite NCs were prepared by the hot-injection method as performed in our previous works.^9–11,22^ Cs_2_CO_3_ (2.50 mmol) was added to a mixture of 1-octadecene (40 mL) and OA (2.5 mL). The mixture was dried for 1 h at 120 °C and then heated under Ar gas to 150 °C. The resulting Cs-oleate precursor solution was preheated to 100 °C.

### 2.3 Synthesis of CsPbBr_3_ NCs

A mixture of 1-octadecene (5 mL) and PbBr_2_ (0.376 mmol) was vacuum dried for 1 h at 120 °C. After purging with Ar gas, OAm (1.0 mL) and OA (1.0 mL) were added to the mixture under stirring at 120 °C, followed by heating to 180 °C. The preheated Cs-oleate precursor (0.8 mL) was rapidly injected to this solution under stirring at 180 °C. After aging for 10 s to obtain CsPbBr_3_ NCs, the resulting dispersion was rapidly cooled in an ice-water bath for 1 min to end the reaction. After adding *tert*-butanol (25 mL), the aggregated NCs were collected by centrifugation at ∼19 000×*g* (13 000 rpm using a rotor with a radius of 10 cm) for 5 min. The supernatant was discarded. After vacuum drying for 24 h, solid sample was obtained. A toluene dispersion was prepared by dispersing the solid sample under stirring and ultrasonication.

### 2.4 Fabrication of green-emitting and red-emitting films

Fluorescent films were prepared using EVA resin.^22^ Granular EVA (1.0 g) was completely dissolved in toluene (10 mL) mixed with 1-octadecene (0.5 mL), OA (28 μL), and OAm (14 μL) under vigorous stirring for 3 h. After Ar gas bubbling at 300 mL min^−1^ for 5 min, the dried NCs sample (20 mg) was dispersed under ultrasonication and stirring for 5 min. A part of the obtained dispersion (3 mL) was dropped onto a Petri dish (∼58 mm bore diameter) and dried for 1 day under ambient conditions to fabricate a green-emitting CsPbBr_3_ NCs nanocomposite film. A red-emitting film was prepared in the same way; a commercial KSF powder (0.40 g) was used instead of the solid sample of CsPbBr_3_ NCs (20 mg). A part of the prepared suspension (8.0 mL) was dried in the Petri dish after ultrasonication and stirring to obtain a red-emitting KSF film. A blank control EVA film without phosphor was also fabricated. The resulting films (thickness: 0.1 mm) were cut into a square (20 mm × 20 mm).

### 2.5 Combination of the films with a blue LED device

Each CsPbBr_3_ NCs film was sandwiched between soda-lime glass plates (20 mm × 20 mm × 1 mm) to shut out the ambient air. Irreversible degradation due to oxidation in the ambient air prevents the self-recovery phenomenon of photodegraded CsPbBr_3_ NCs.^9–11,22^ Adhesive aluminum tape was wrapped around the edges to secure the glass plates. The film samples were placed on a flat-panel blue LED (BTE-4556, Nissin Electronics) equipped with a power supply (LPR-10W, Nissin Electronics) and irradiated for 72 h by turning the device on. The luminescence wavelength and irradiance of used blue LED were 468 nm and 48.5 W m^−2^, respectively. After the irradiation was stopped, they were stored in the dark without separation. The blue LED was temporarily turned on when photography and measurements were needed.

### 2.6 Characterization

The particle morphologies were observed using a field-emission transmission electron microscope (Tecnai G^2^, FEI). Transmission electron microscopy (TEM) sample was prepared by vacuum drying a drop of NCs dispersion on carbon-reinforced collodion-coated copper grids (COL-C10, Oken Shoji) overnight. X-ray diffraction (XRD) profiles were acquired with an X-ray diffractometer (MiniFlex600, Rigaku) equipped with a Cu Kα radiation source. FT-IR absorption spectra of dried NCs samples in pressed KBr disks were recorded using an FT-IR spectrometer (FT/IR-4200, JASCO) under an N_2_ gas flow of 400 mL min^−1^. UV-vis absorption spectra were measured using UV/visible/near-infrared optical absorption spectrometers (V-750 and V-570, JASCO). Here, integrating sphere (ISF-513, JASCO) was used for film samples to obtain total transmittance and absorbance. Tauc plots were prepared according to the eqn (1) to determine the *E*_g_ values of the NCs.S1
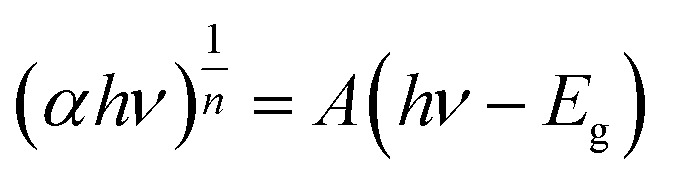
where *α* is absorbance, *h* is the Planck constant, *ν* is frequency, and *A* is a constant. The value of *n* was 0.5 because CsPbBr_3_ is a direct transition-type semiconductor. PL spectra were measured using fluorescence spectrometers (FP-6500 and FP-8500, JASCO). The obtained spectra were calibrated. Absolute PL quantum yield (PLQY) was determined using a quantum efficiency measurement system (QE-2000-311C, Otsuka Electronics). Emission spectra of the LED devices were measured by an LED evaluation system (Hamamatsu Photonics, C9920-22).

## 3 Results and discussion

### 3.1 Fundamental evaluation of the synthesized CsPbBr_3_ NCs

The average size of the as-prepared CsPbBr_3_ NCs was 8.7 ± 1.7 nm (see Fig. S1†). They had cubic crystal structure and were modified by oleate anions and oleylammonium cations (see Fig. S2†). The resulting NCs dispersed in toluene had 2.43 eV of band gap and showed green luminescence under UV excitation (see Fig. S3†). The band gap was larger than 2.00 eV of bulk cubic CsPbBr_3_,^1^ indicating the quantum size effect.

### 3.2 Change in emission color of the LED device attached with the green-emitting film through the photodegradation and self-recovery of CsPbBr_3_ NCs

**Fig. 1:**
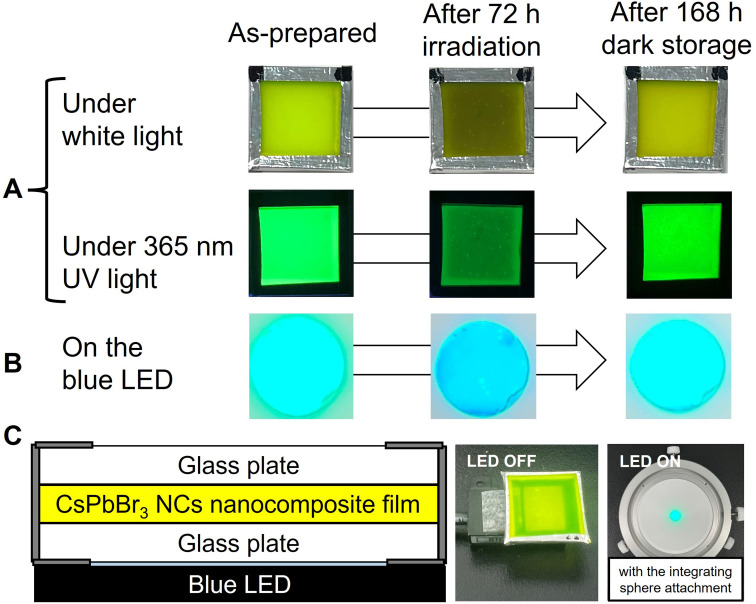
(A) Changes in sample color under white light and luminescence under UV excitation for the CsPbBr_3_ NCs nanocomposite film after 72 h blue LED irradiation and subsequent 168 dark storage. (B) Emission of the film irradiated with the 468 nm blue LED is also shown. (C) Illustration of the cross-sectional structure and photographs of the settings.

As illustrated in Fig. 1, to prevent air oxidation for CsPbBr_3_ NCs in EVA, the green-emitting nanocomposite film cut into 20 mm squares was sandwiched between glass plates and fixed with aluminum tape. It was placed on a 468 nm blue LED device (48.5 W m^−2^). The nanocomposite film was irradiated for 72 h and then stored in the dark for 168 h. As shown in the photographs, blackening of the NCs nanocomposite films occurred during the blue LED illumination, which was accompanied by a decrease in PL intensity. Increase in the PL intensity was observed during storage in the dark. In our previous works, the change in adsorption state of the surface ligands was evaluated by FT-IR analysis on dried solid NC sample.^9,11^ Unfortunately, effective analysis could not be performed due to the low concentration of NCs in the present nanocomposite film. The luminescence of the NCs nanocomposite film placed on the blue LED was also captured. Blue-green light was observed, which was a mixture of green PL of excited NCs and transmitted blue light. The mixed light intensity exhibited decrease and increase in the same manner. Its color also changed to the naked eye, although it is difficult to recognize in the photographs due to performance limitations of the used camera.

**Fig. 2:**
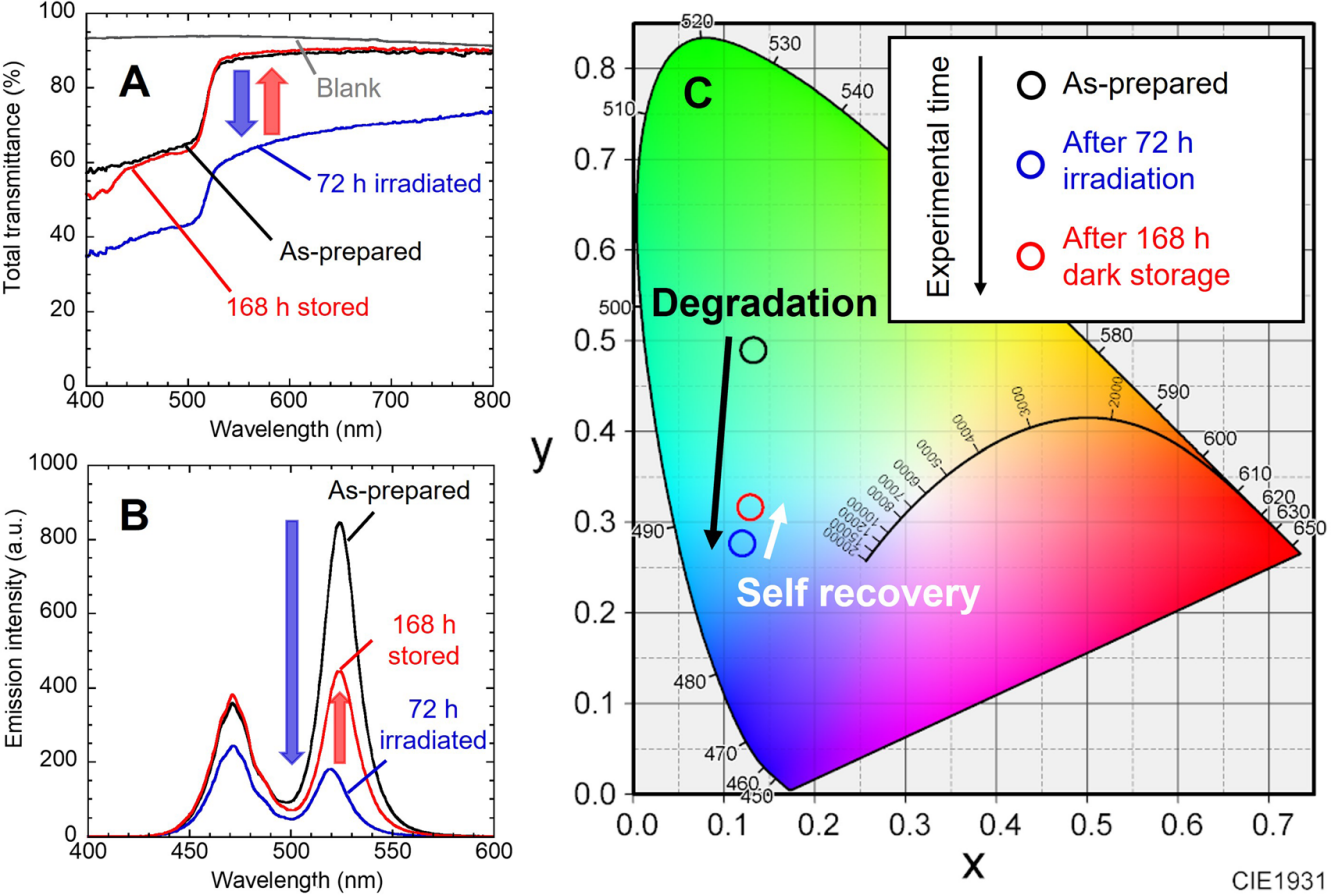
(A) Change in transmission spectrum for the CsPbBr_3_ NCs nanocomposite film. (B) Change in emission spectrum of the nanocomposite film on the 468 nm blue LED device. (C) Corresponding chromaticity coordinates of the emission.

Fig. 2A shows the change in the total light transmission spectrum of the individual nanocomposite film. Blue light irradiation caused a decrease in transmittance in the whole wavelength region. No change was observed in the position of absorption edge attributed to the interband transition of NCs. After storage in the dark, the transmittance returned to almost the initial state. These changes reproduced the results of our previous study.^22^ The change in emission spectrum is also shown (Fig. 2B). The decrease in blue emission at 468 nm is attributed to increased absorption of the nanocomposite film becoming black. The decrease in green emission is due to photodegradation of the NCs caused by the photo-induced desorption of surface ligands. After the LED was turned off and stored in the dark for 168 h, the emission intensity naturally increased. The blue light intensity also recovered to the initial value as the film color returned to its initial state. On the other hand, the green PL intensity recovered to only half of its initial value. This is presumably due to the irreversible degradation of NCs gradually progressed by residual oxygen and water molecules in the film during the 10 days experiment.^22,23^ Degradation occurs without excitation light (see Fig. S4†). When the nanocomposite film was stored in the dark, natural degradation was observed. Its absorption spectrum was mostly preserved, while its PL intensity decreased to 43% of the initial intensity after the dark storage for 10 days. The peak position of green PL shifted from 524 nm to 519 nm by light irradiation, and then returned to 524 nm after storage in the dark. These indicate the desorption and re-adsorption of the surface ligands. The synthesized CsPbBr_3_ NCs are quantum dots, as indicated by the quantum size effect described earlier. The band gap of quantum dots is known to change due to the influence of surface ligands,^24^ and it has been reported that the band gap of CsPbI_3_ NCs also changed due to surface ligands.^25^ Therefore, the shifts of the PL peak suggest changes in the adsorption state of the surface ligands. Moreover, the NCs in nanocomposite film are surrounded by EVA resin. EVA, which has ester group (–COO–), possibly affect the photodegradation and self-recovery through adsorption to the NC surface. Unfortunately, effective analysis could not be performed due to the low concentration of NCs in the nanocomposite film. The chromaticity coordinates shifted in accordance with the change in the emission spectrum (Fig. 2C). While the blue LED was turned on, the emission changed from green to blue. After the light was turned off, the color shifted toward green. If the resin shut out oxygen and moisture, a complete self-recovery of the PL properties of the NCs should occur,^9^ resulting in a color shift closer to the initial one.

### 3.3 Change in emission color of the LED device attached with the red-emitting film and green-emitting film through the photodegradation and self-recovery

**Fig. 3:**
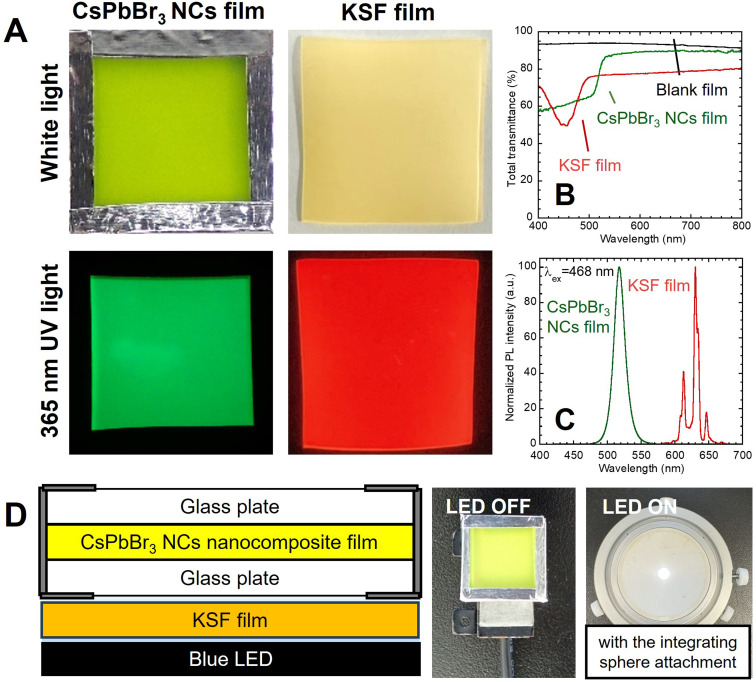
(A) Photographs under white light and UV excitation, (B) transmission spectra, and (C) PL spectra of the CsPbBr_3_ NCs nanocomposite film and KSF film used in a white LED device. (D) Illustration of the cross-sectional structure and photographs of the settings.

The red-luminescent film was prepared by dispersing the commercial KSF phosphor to the EVA resin. As shown in Fig. 3A, the KSF film showed a light-yellow color under white light and red PL under UV light excitation. The transmission spectrum showed an absorption peak of KSF phosphor at 468 nm (Fig. 3B), while the PL spectrum showed multiple sharp peaks due to Mn^4+^ at 600–650 nm (Fig. 3C).^20,21^ As shown in the illustration (Fig. 3D), a white LED device was constructed by overlaying the nanocomposite film and the KSF film onto the blue LED. It should be noted that the nanocomposite film was placed uppermost to directly observe the color change without disassembly.

**Fig. 4:**
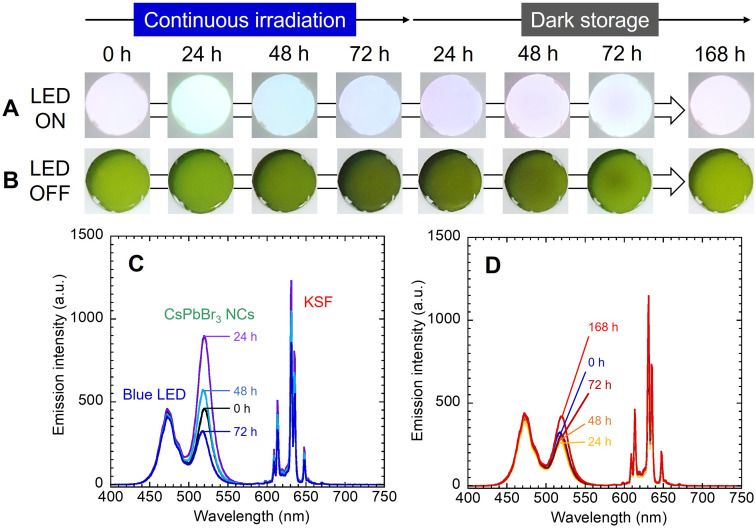
(A) Change in emission color of the white LED composed of the CsPbBr_3_ NCs nanocomposite film, KSF film, and blue LED. (B) Photographs under white light with the blue LED turned off are also shown. Corresponding change in emission spectrum during (C) the continuous irradiation and (D) the subsequent dark storage.

**Fig. 5:**
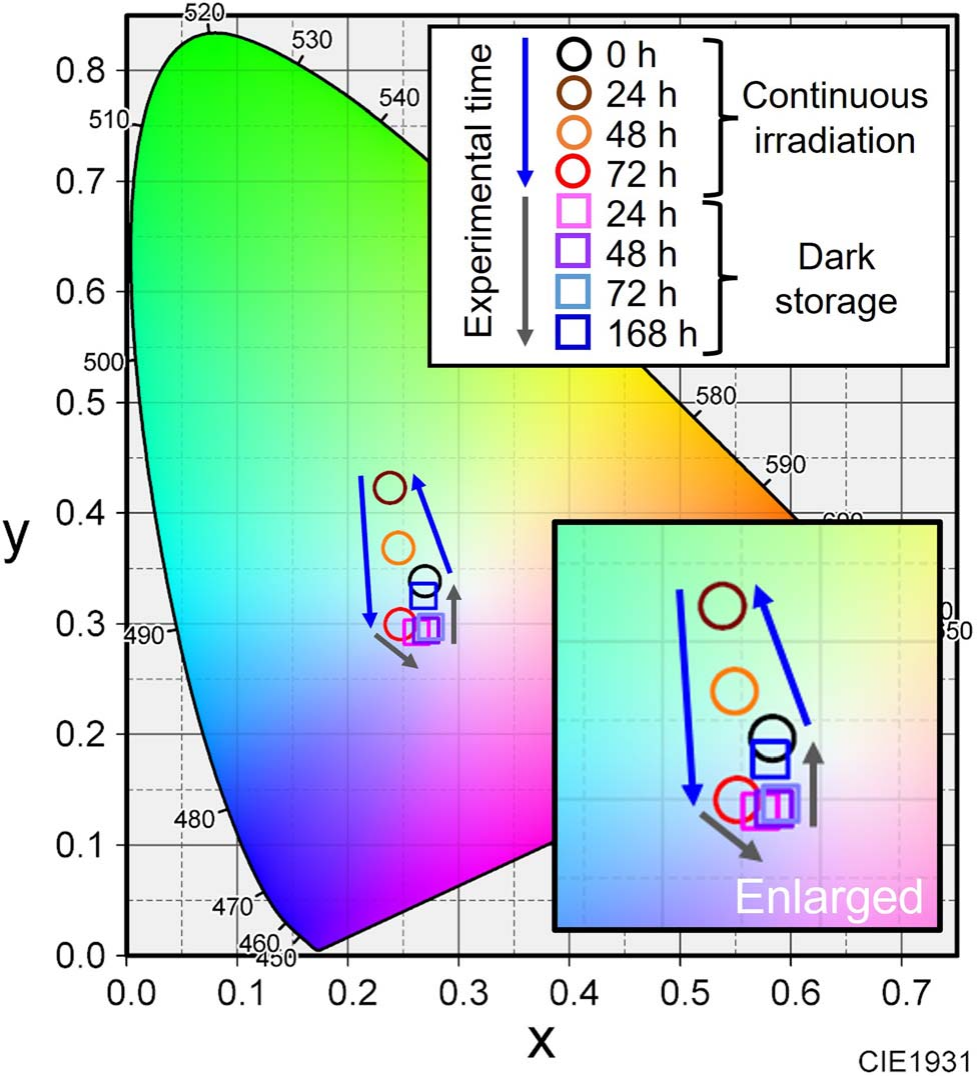
Change in color chromaticity corresponding to the emission spectrum in Fig. 4.

These films were fixed to the integrating sphere attachment, and the LED was turned on continuously for 72 h, and then turned off and stored in the dark for 168 h. The LED was temporarily turned on and off when necessary for photography and emission spectrum measurements. As shown in Fig. 4 and 5, the observed light became greenish white after 24 h of exposure to blue light and bluish white after 72 h. After 48 h of storage in the dark, the light color turned reddish, and after 72 h, it became bluish again. After 168 h, it showed the same white illumination as the initial state; the chromaticity coordinate of the last emission was located near the first coordinate. In the photographs when the LED was turned off, the nanocomposite film turned black by blue light irradiation and returned to its original color during subsequent storage in the dark.

In the emission spectrum of the white LED device (Fig. 4C), the emission peaks of the blue LED, CsPbBr_3_ NCs and KSF were observed at 472 nm, 519 nm and 631 nm, respectively. A decrease in the intensity of the blue and red peaks was observed during the LED irradiation (see also Fig. S5†), caused by increased absorption by the blackened film. The green peak intensity temporally increased up to 24 h after blue light irradiation and then decreased. The PL enhancement would be attributed to the photo-activation caused by optimization of the adsorption states of the surface ligands.^26,27^ This phenomenon should occur in competition with the photo-induced desorption of the surface ligands. When the excitation light intensity is sufficiently strong, the photo-induced desorption becomes dominant and only degradation of PL properties is observed as described earlier. On the other hand, in this experiment, partial blue light transmitted through the KSF film was irradiated onto the nanocomposite film. In other words, the nanocomposite film was irradiated with blue light that was weaker than that directly irradiated from the LED. The PL enhancement due to the photo-activation phenomenon was probably observed as a result of the slower progression of the photo-induced desorption. The increase was temporary, as influence of the photo-induced desorption became dominant thereafter. The green peak intensity continued to decrease immediately after the blue LED was turned off (Fig. 4D). However, it began to increase 24 h after the dark storage began. Slow spontaneous degradation due to residual oxygen and moisture in the film should progress during the experiment. The effect of re-adsorption of surface ligands increased gradually, and self-recovery was observed after a while. The intensity of the blue and red emission peaks also increased as the color of the nanocomposite film returned to the initial color during dark storage (see Fig. S5†).

## 4 Summary

The demonstration of photochromic PL switching shown by this work would expand the potential applications of photodegradation and self-recovery of CsPbBr_3_ NCs. The current major problem is irreversible degradation due to oxygen and moisture.^22,23^ In order to realize the reversible phenomena, it will be necessary to prevent serious irreversible damage to CsPbBr_3_ NCs from the external environment.^8,10,28–30^ Another current problem is controlling the speed of recovery, as the full recovery took several months.^9^ After solving the problem, the feasibility of entering new applications in the fields of nanomaterials and LED technology will be demonstrated in the future.

## Data availability

The data supporting this article have been included as part of the ESI.†

## Conflicts of interest

There are no conflicts to declare.

## Supplementary Material

RA-014-D4RA04567J-s001
