# Supplementary material for: Implications for applicability of the photodegradation and self-recovery of green-emitting CsPbBr3 perovskite nanocrystals
Source: RSC Adv. 2024 Aug 19;14(36):26059–65. doi: 10.1039/d4ra04567j (PMC11332588; doi:10.1039/d4ra04567j)
Supplement: RA-014-D4RA04567J-s001 [file RA-014-D4RA04567J-s001.pdf]

Supplementary Information (SI) for RSC Advances.
This journal is © The Royal Society of Chemistry 2024

Electronic Supplementary Information

RSC Advances

Implications for Applicability of the Photodegradation and Self-Recovery of Green-Emitting $CsPbBr_3$ Perovskite Nanocrystals

Yoshiki Iso*, Shunsuke Saito, Hikari Toyoda, and Tetsuhiko Isobe*

Department of Applied Chemistry, Faculty of Science and Technology, Keio University, 3-14-1 Hiyoshi, Kohoku-ku, Yokohama 223-8522, Japan

*Corresponding authors.

Yoshiki Iso – E-mail: iso@applc.keio.ac.jp; Tel.: +81 45 566 1558; Fax: +81 45 566 1551; orcid.org/0000-0001-7483-2828

Tetsuhiko Isobe – E-mail: isobe@applc.keio.ac.jp; Tel.: +81 45 566 1554; Fax: +81 45 566 1551; orcid.org/0000-0002-0868-5425

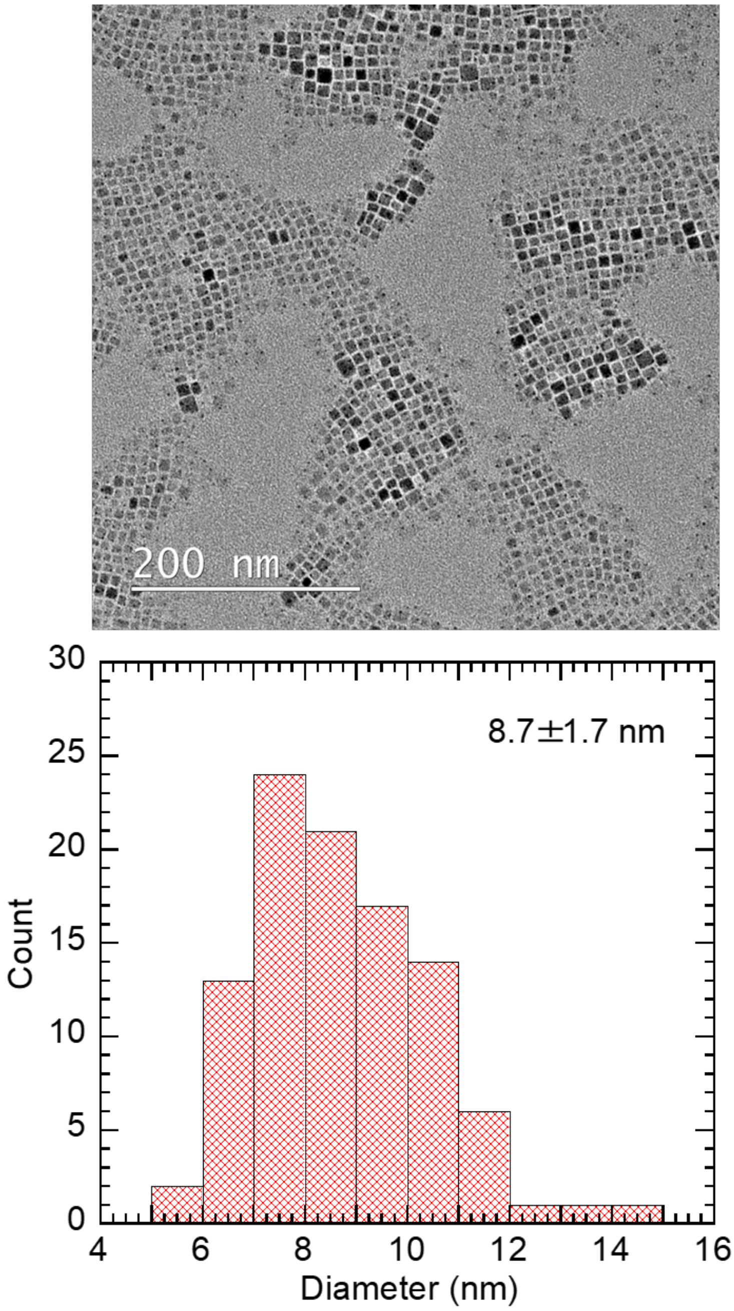

Fig. S1 TEM image of the as-prepared $CsPbBr_3$ NCs.

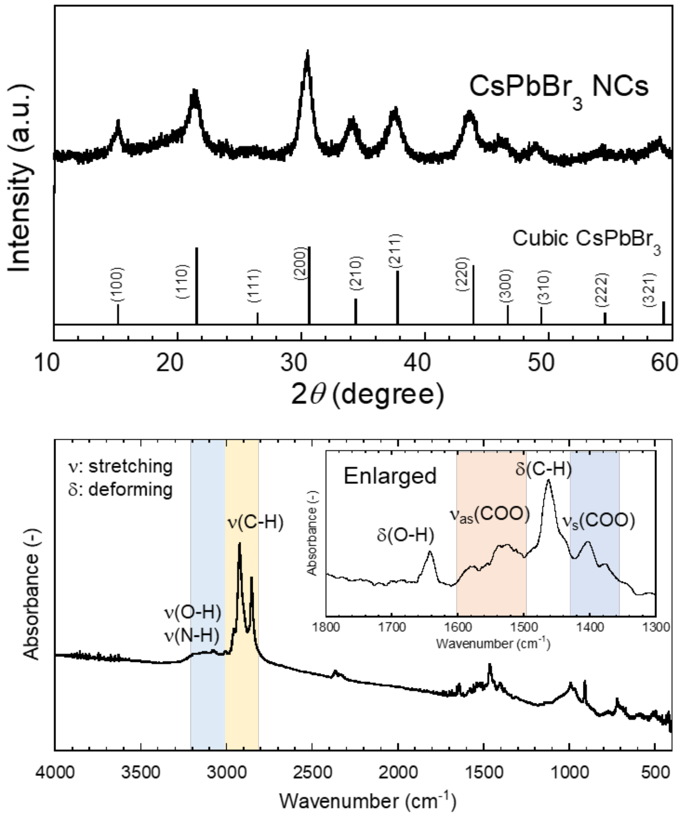

Fig. S2 XRD profile and FT-IR spectrum of the as-prepared $CsPbBr_3$ NCs. ICDD card data of cubic $CsPbBr_3$ (no. 00-054-0752) is also shown. The obtained sample is assigned to cubic $CsPbBr_3$ NCs by the XRD result. The FT-IR spectrum confirms surface ligands on the NCs.[S1] The absorption peaks assigned to $\nu_{as}(COO^-)$ and $\nu_s(COO^-)$ reveals adsorption of oleate ion on the NC surface. The broad peak at ~3100 cm^{-1} can be attributed to ν(NH) of oleylammonium ion. It should be noted that the peak of δ(OH) at ~1640 cm^{-1} indicates adsorption of H_2O from the ambient air. The peak of ν(NH) would be overlapped with that of ν(OH). The peaks of ν(CH) and δ(CH) are derived from oleyl group in oleate ion and oleylammonium ion.

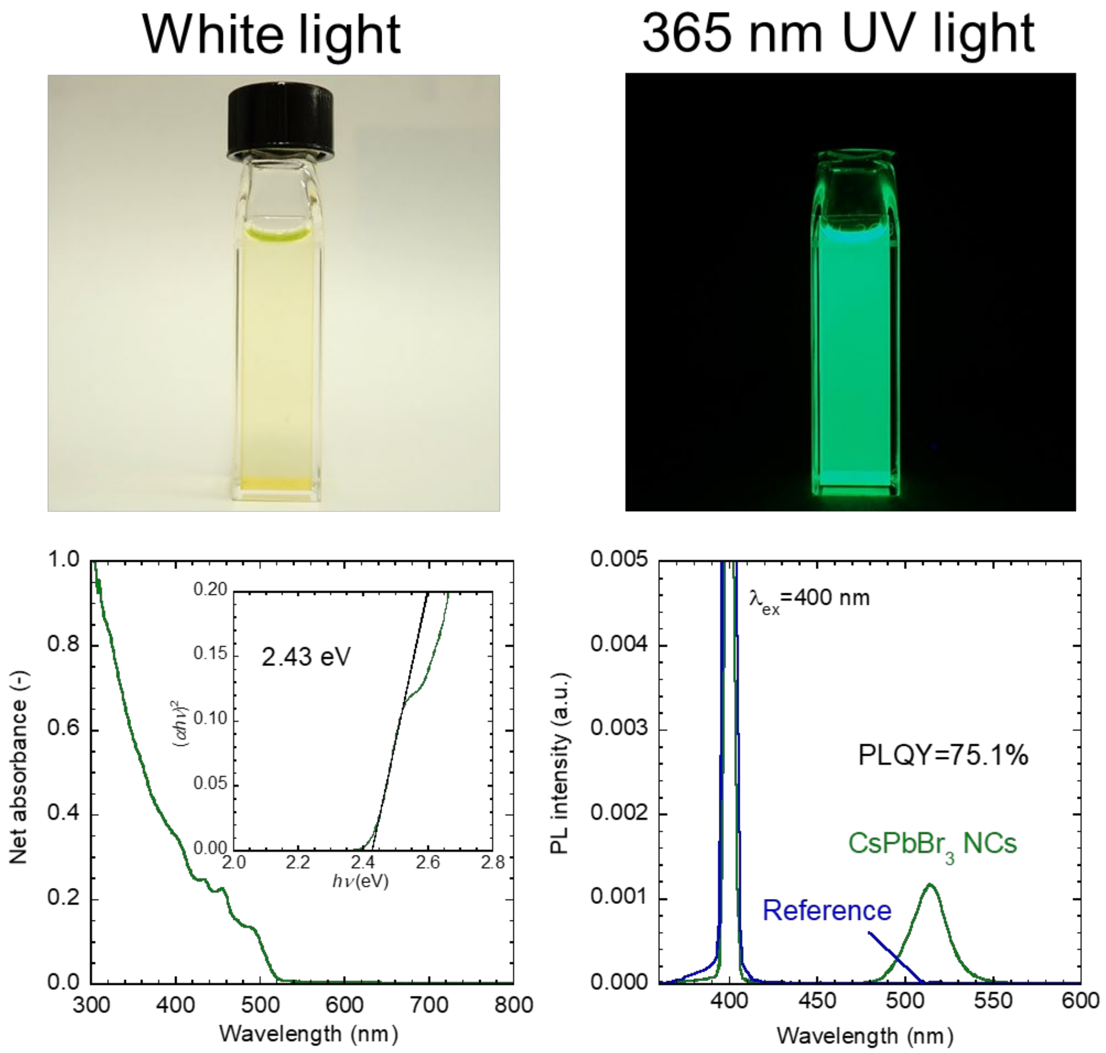

Fig. S3 Photographs, UV-vis spectrum, Tauc plot (inset), and PL spectrum of as-prepared $CsPbBr_3$ NCs dispersed in toluene.

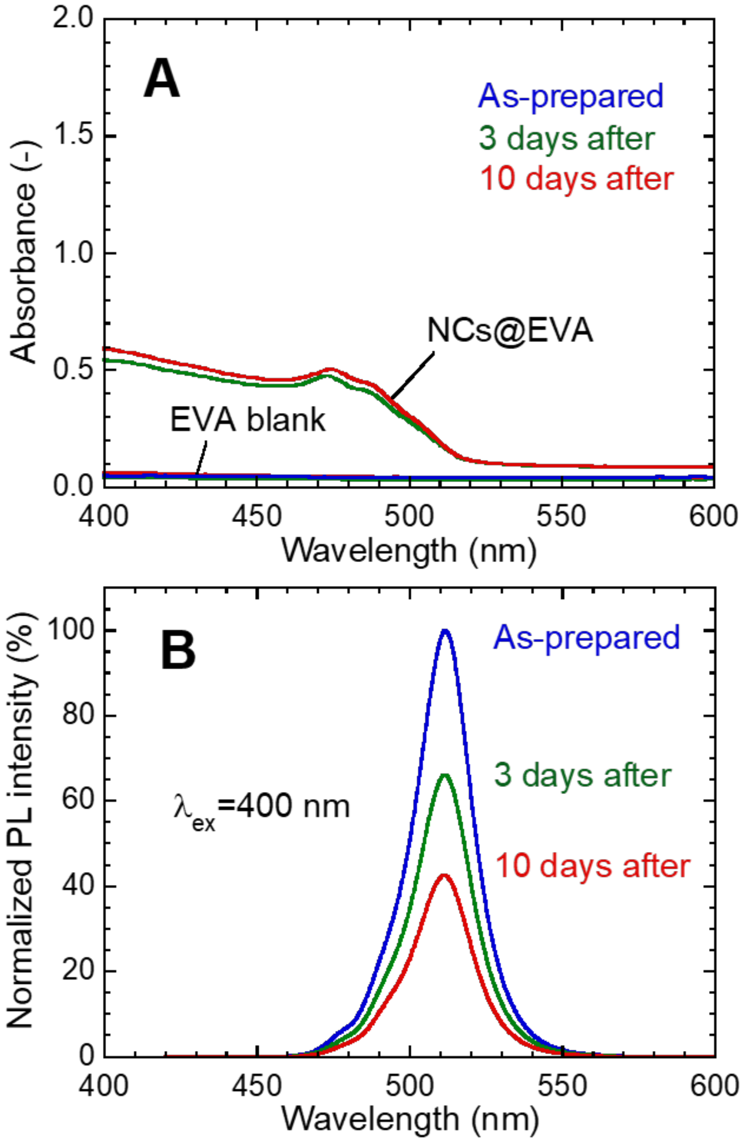

Fig. S4 Changes in (A) UV-vis absorption and (B) PL spectra of $CsPbBr_3$ NCs film during dark storage. The absorption spectrum was mostly preserved, while the PL peak intensity decreased to 66% and 43% to the initial intensity after the dark storage for 3 and 10 days, respectively.

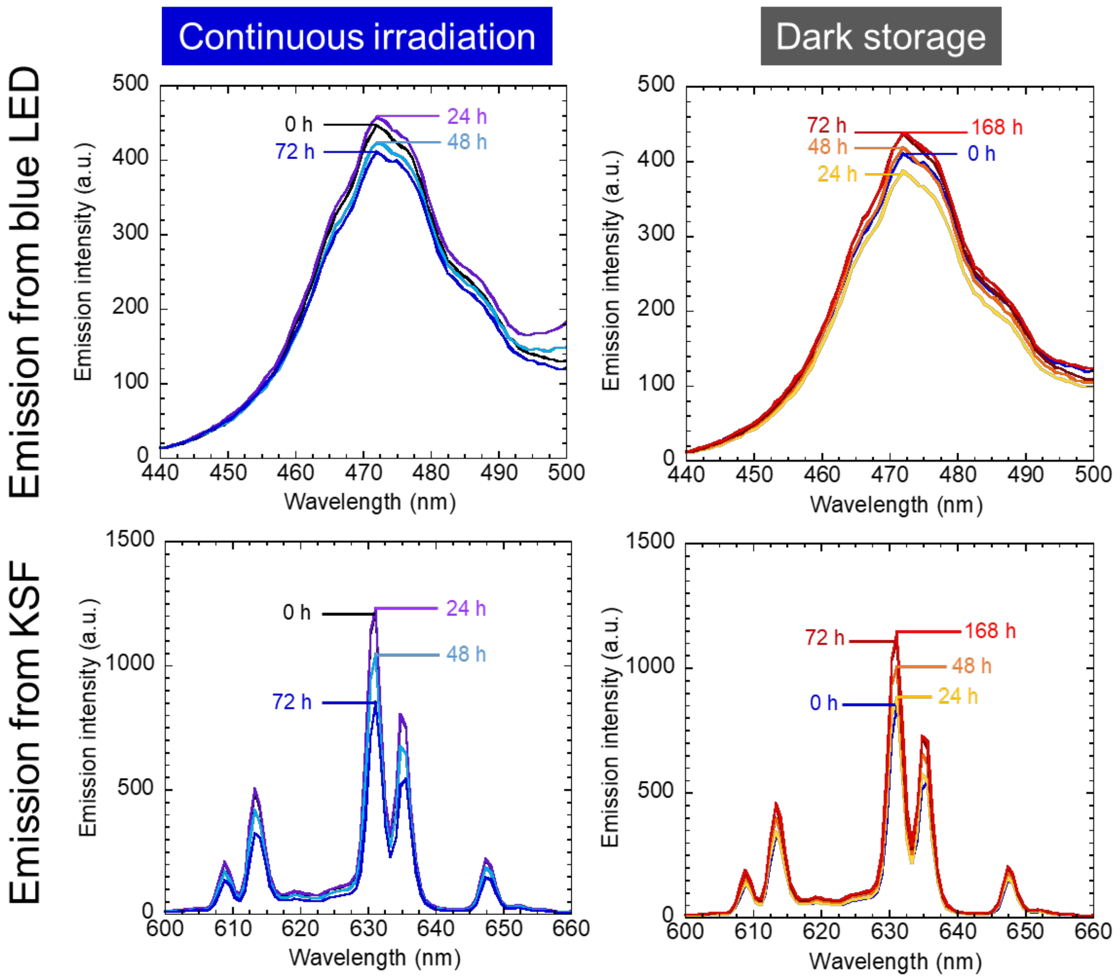

Fig. S5 Changes in emission intensity of the blue peak and the red peaks during continuous blue LED irradiation and subsequent dark storage extracted from the emission spectra of Fig. 4.

Reference

S1) P. J. Larkin, *Infrared and Raman Spectroscopy: Principles and Spectral Interpretation*, Elsevier, Amsterdam, 2nd edn, 2017, pp. 85–134.
